# Phylogenomics Reveals the Evolutionary History of *Phytolacca* (Phytolaccaceae)

**DOI:** 10.3389/fpls.2022.844918

**Published:** 2022-06-10

**Authors:** Yun Song, Fan Jiang, Junxia Shi, Chaonan Wang, Ning Xiang, Shuifang Zhu

**Affiliations:** Institute of Plant Inspection and Quarantine, Chinese Academy of Inspection and Quarantine, Beijing, China

**Keywords:** biogeography, dispersal events, *phytolacca*, phylogenomics, chloroplast genome

## Abstract

*Phytolacca* is the largest genus of Phytolaccaceae. Owing to interspecific hybridization, infraspecific variation, and apparent weak genetic control of many qualitative characters, which have obscured boundaries between species, the classification and phylogenetic relationships of this genus are unclear. Native *Phytolacca* is disjunctly distributed in America, eastern Asia, and Africa, and the biogeographic history of the genus remained unresolved. In this study, we used the whole chloroplast genome and three markers (nrDNA, *rbcL*, and *matK*) to reconstruct phylogenetic relationships within *Phytolacca*, analyze divergence times, and infer biogeographic histories. The phylogenetic results indicate that *Phytolacca* is monophyletic, which is inconsistent with the infrageneric classification based on morphology. According to the divergence time estimation, *Phytolacca* began to diversify at approximately 20.30 Ma during the early Miocene. Central America, including Mexico, Costa Rica, and Colombia, is the center of species diversity. Biogeographical analysis indicated five main dispersal events and *Phytolacca* originated from Central and South America. Birds may be the primary agents of dispersal because of the fleshy fruiting of *Phytolacca*. This study extended sampling and added more genetic characteristics to infer the evolutionary history of *Phytolacca*, providing new insights for resolving the classification and elucidating the dispersal events of *Phytolacca.*

## Introduction

Phytolaccaceae *sensu lato* (*s.l.*) includes 17 genera and approximately 70–80 species (Nowicke, 1968; Rohwer, 1993) and comprises weedy and polyphyletic genera (Schäferhoff et al., 2009; Lee et al., 2013). This family has been disentangled step by step over the last decades, and Phytolaccaceae *sensu stricto* (*s.s*.) includes three genera (*Anisomeria, Ercilla*, and *Phytolacca*) (The Angiosperm Phylogeny Group, 2016; Yao et al., 2019). Phytolaccaceae *s.s.* is actually the subfamily of Phytolaccoideae, which possesses multiple carpels per flower. Their carpels are united or free, and fruits are drupe, achene or berry (Nowicke, 1968; Rohwer, 1993).

*Phytolacca* L. is commonly known as ‘pokeweed’. Species of *Phytolacca* are perennial herbs, shrubs and trees; the stems are green, pink or red; and the fruits are defined as berry, green at first but dark purple to black after ripening (Nowicke, 1968; Rogers, 1985; Caulkins and Wyatt, 1990; Vanvinckenroye and Smets, 1997). *Phytolacca* is the largest genus in the family Phytolaccaceae *s.s.*, with the number of species ranging from 20 (Nowicke, 1968) to over 35 (Willis, 1966). Most *Phytolacca* species contain phytolaccatoxin and phytolaccigenin, and the active principles have been reported to be effective for analgesic, anti-inflammatory, bactericidal, fungicidal, mitogenic and molluskicide action (Choe et al., 2020), meanwhile, *Phytolacca* species are toxic plants (Monkiedje et al., 1991; Xiao et al., 2022). The berries and young sprouts and leaves of some species of *Phytolacca* are used as adulterants of red wine and poke salad, respectively (Nowicke, 1968). Several species, including *P. dioica* L., are cultivated as shade trees in the tropics. *Phytolacca americana* L. and *P. acinosa* Roxb. occasionally escape and become naturalized (Bentley et al., 2015).

The taxonomy of *Phytolacca* species remains confusing, and phylogenetic relationships are not resolved because of the common occurrence of intraspecific variability and hybridization (Fassett and Sauer, 1950; Rogers, 1985; Caulkins and Wyatt, 1990; Rohwer, 1993). According to the degree of connation of the carpels, *Phytolacca* was classified into three subgenera (Nowicke, 1968): *Pircunia* (carpels completely free), *Pircuniopsis* (carpels more or less united) and *Phytolacca* (carpels completely united, styles more or less connivent). Each subgenus was further divided into two sections based on the characteristics of flowers. Namely, the subgenus *Pircunia* was divided into two sections, *Pircunia* (flowers perfect) and *Pircunioides* (flowers pistillate or staminate); the subgenus *Pircuniopsis* was divided into two sections, *Pircuniophorum* (flower perfect) and *Pircuniopsis* (flowers pistillate or staminate); and *Phytolacca* was divided into two sections, *Phytolacca* (flower perfect) and *Phytolaccoides* (flowers pistillate or staminate). Interspecific hybridization, infraspecific variation, and apparent weak genetic control of many qualitative characters have obscured boundaries between species (Nowicke, 1968). The number of species ranged from 20 (Nowicke, 1968) to over 35 (Willis, 1966). In recent years, several new species have been published based on their morphological characteristics (Xie et al., 2017; Li et al., 2020). Only one study used ITS data to infer the phylogeny of *Phytolacca* (Ali et al., 2015), showing the relationships among the species did not show harmony with the infrageneric classification based on morphology. The ITS tree-supported *Phytolacca* species were divided into three clades (Ali et al., 2015).

Native *Phytolacca* species are distributed mostly from southeastern Canada, southward Central and South America, and in the West Indies. In the Old World, a small number of species range from Africa and Madagascar into Asia Minor and eastward across southern Asia to Korea, Japan, and China (Rogers, 1985). To better understand the historical dispersal of *Phytolacca* and the role of dispersal in shaping the overall disjunct patterns, it is essential to infer the historical biogeography and reveal the times of the *Phytolacca* dispersal events.

A robust species phylogeny is essential to understand the evolutionary history of a plant group, but the phylogenetic relationships of most species of *Phytolacca* remain unresolved due to the low divergence and insufficient phylogenetic information of the ITS (Ali et al., 2015). To reveal the evolutionary history of *Phytolacca*, it is essential to add more molecular data and reconstruct a highly resolved species tree. Chloroplast genome sequences provide effective genetic markers to resolve complex evolutionary histories (Dong et al., 2022a,b). Meanwhile, chloroplast genomes are mostly inherited uniparentally, lack recombination, and have a compact size. Although the genome structure is conserved, mutational events, including indels, SSRs, and single nucleotide substitutions (SNPs), frequently occur even in related species (Dong et al., 2021a,b).

To better resolve the relationships, divergence time, and historical dispersal of *Phytolacca*, we sequenced the chloroplast genome and nrDNA (nuclear ribosomal DNA) of *Phytolacca* species and added published molecular data from GenBank. Specifically, we attempted to (1) investigate the relationships within *Phytolacca*, (2) estimate the divergence time of *Phytolacca*, and (3) elucidate historical dispersal events within *Phytolacca.* This study also sheds new light on transcontinental dispersal routes.

## Materials and Methods

### Plant Material and DNA Sequencing

We collected fresh healthy leaves from 17 individuals representing 9 species of *Phytolacca* and comprising six samples of *P. americana*, two samples of *P. acinosa* and *P. dioica*, and one each of the remaining six species. Six samples were sampled from the Plant DNA Bank of China at the Institute of Botany, Chinese Academy of Sciences. The details of the samples are presented in Supplementary Table 1. The chloroplast genomes of *P. insularis* (MH376309) and *P. americana* (MH286315) are available in GenBank, and we added them to our analyses. To infer the complete phylogeny of the genera, we added published molecular data from GenBank (Supplementary Table 2), including three genes (nrDNA, *rbcL*, and *matK*).

We performed extractions of the total genomic DNA *via* a modified cetyltrimethylammonium bromide (CTAB) method (Li et al., 2013) and assessed the DNA quality and concentration with agarose gel electrophoresis. The total DNA was fragmented to 350 bp to construct a library for sequencing on an Illumina HiSeq X-ten at Novogene (Tianjin, China). Each sample yielded approximately 5 Gb of high-quality 150-bp paired-end reads.

### Genome Assembly and Annotation of Chloroplast Genome and Nuclear Ribosomal DNA

Raw reads were assessed for quality with Trimmomatic 0.39 (Bolger et al., 2014) with the following parameters: LEADING = 20, TRAILING = 20, SLIDING WINDOW = 4:15, MIN LEN = 36, and AVG QUAL = 20. Whole chloroplast genomes and nrDNA were accomplished utilizing GetOrganelle (Jin et al., 2020), with a k-mer length of 85 bp. The correctness of the assembly was confirmed by using Geneious to map all clean reads to the assembled complete chloroplast genome. All chloroplast genomes were annotated by using Perl script Plann (Huang and Cronk, 2015) with a reference genome (*P. insularis*, GenBank: MH376309). Furthermore, the annotations with problems were manually edited by using Sequin. A circular diagram for the chloroplast genome was generated using OGDRAW (Greiner et al., 2019), and the complete chloroplast genome and nrDNA sequences have been deposited in GenBank.

### Comparative Chloroplast Genome Analysis

We compared the chloroplast genomes of ten *Phytolacca* species: the nine newly sequenced in this study and the previously published chloroplast genome of *P. insularis*. The Perl script MISA^1^ was used to exploit simple sequence repeats (SSRs). The minimum number of repeats was ten for mono, five for di-, four for tri-, and three each for tetra-, penta, and hexanucleotide SSRs.

The alignment of the whole chloroplast genome was retrieved by MAFFT (Katoh and Standley, 2013). In order to obtain an accurate aligned sequence matrix, we examined and adjusted manually, for examples, the erroneous alignments in the polymeric repeat structures and small inversion structures to avoid amplifying sequence divergence. Based on the accurate aligned sequence matrix, the genetic p-distance among the ten *Phytolacca* species was calculated using MEGA 7.0 (Kumar et al., 2016). To explore highly variable chloroplast markers, we used the sliding window method to calculate nucleotide diversity (π) by DnaSP v6 (Rozas et al., 2017) with a window size of 600 bp and a step size of 50 bp.

The number of variable sites and nucleotide diversity were used to assess marker variability for hypervariable markers. The three universal chloroplast DNA barcodes *rbcL*, *matK*, and *trnH-psbA* were used in this analysis.

### Phylogenetic Analyses

To understand the interspecific phylogenetic relationship of *Phytolacca* and the phylogenetic position of Phytolaccaceae *s.s.* in Caryophyllales, three datasets were used for phylogenetic analysis. The first dataset (19 cpg) was the 18 *Phytolacca* chloroplast genome with *Ercilla volubilis* as an outgroup. The second dataset (83g48s) contained 79 coding genes and four rRNA genes, including 18 *Phytolacca* samples and 30 Phytolaccaceae *s.l.* and its allied species (Supplementary Table 3). The third dataset (3g22s) contained three markers, nrDNA, *rbcL* and *matK*, including 22 *Phytolacca* species and three outgroups (*Agdestis clematidea*, *Sarcobatus vermiculatus*, and *Ercilla volubilis*). Coding and rRNA genes were extracted using Geneious Prime v2020.0.5 based on the annotation of the chloroplast genomes.

Two methods, maximum likelihood (ML) and Bayesian inference (BI), were used for phylogenetic analyses. RAxML-NG (Kozlov et al., 2019) was used to perform the ML analysis with 500 replicates and the best-fit model from ModelFinder (Kalyaanamoorthy et al., 2017). BI analysis was conducted using Mrbayes v3.2 (Ronquist et al., 2012) with the nucleotide substitution model inferred from ModelFinder (Kalyaanamoorthy et al., 2017). Markov chain Monte Carlo (MCMC) analyses were run for 20 million generations with sampling over 100 generations. The MCMC convergence was determined by calculating the average standard deviation of split frequencies, which fell below 0.01. The stationary phase was examined through Tracer 1.6 (Rambaut et al., 2014) and the first 25% of the sampled trees was discarded. The remaining trees generated a majority-rule consensus tree to estimate posterior probabilities.

### Divergence Time Estimation

BEAST v2.5.1 (Bouckaert et al., 2014) was used to estimate the divergence times of Phytolaccaceae *s.l.* and its allies using five priors based on the 83g48s dataset. The pollen fossils from the mid-Eocene (41.2 Ma) of Argentina assigned it to Nyctaginaceae (genus is indeterminate) based on its morphological similarity compared with that of the extant taxa of the family (Zetter et al., 1999). This fossilized pollen was used to offset the crown of Nyctaginaceae and was given a lognormal distribution with offset values as specified (41.2 Ma, with a mean of 1.5 and a standard deviation of 1), allowing for the possibility that the crown of Nyctaginaceae is considerably older than the fossils themselves. According to the average value obtained by Yao et al. (2019) in a calibrated analysis, four priors were used: (i) the average age of the most recent common ancestor (MRCA) of the Phytolaccoid clade (including Agdestidaceae, Nyctaginaceae, Petiveriaceae, Phytolaccaceae, Sarcobataceae) was 62.4 Ma; (ii) the stem age of Aizoaceae (the root of the tree) was 79.7 Ma; (iii) the split between Agdestidaceae and Sarcobataceae was 47.1 Ma; and (iv) the crown age of Phytolaccaceae *s.s.* was 25.7 Ma. Each secondary prior was placed under a normal distribution with a standard deviation of 1.

Furthermore, we used the 3g22s dataset to infer the divergence time at the species level. Three priors from the above results were used for this analysis: (i) the crown age of Phytolaccaceae *s.s.* and Sarcobataceae/Agdestidaceae (the root of the tree); (ii) the crown age of Phytolaccaceae *s.s.*; (iii) the crown age of *P. dioica* and its sister group. All three priors were placed under a normal distribution with a standard deviation of 1.

The GTR model and the prior tree Yule model were selected with the uncorrelated lognormal distribution relaxed molecular clock model. The MCMC analysis ran for 500,000,000 generations with sampling every 10,000 generations. The stationary phase was examined through Tracer 1.6 (Rambaut et al., 2014) to evaluate convergence and to ensure sufficient and effective sample size (ESS) for all parameters surpassing 200. The first 10% of the trees was discarded as burn-in, and then the MCC tree was determined with mean heights in TreeAnnotator.

### Biogeography of *Phytolacca*

The distribution area of each species was determined using specimen records and literature data (Borak Martan and Šoštarić, 2016; Xie et al., 2017; Li et al., 2020). Georeferenced specimen records were obtained from the Global Biodiversity Information Facility (GBIF)^2^ and the National Specimen Information Infrastructure of China (NSII).^3^ According to the database of Plants of the World Online,^4^ we checked the distribution records and corrected for potential errors as a result of typographical errors or introduced plant records.

To construct the distribution patterns of species diversity, we divided countries that are larger than 200,000 km^2^ into smaller units (e.g., into official administrative units of each country, such as provinces or states). For each of these administrative units, we examined and recorded the number of *Phytolacca* species within them.

For biogeographic reconstructions, taxa of *Phytolacca* were assigned to four areas based on their present distributions: Africa, Central and South America, Eastern Asia and North America. We used the 3g22s dataset to infer the ancestral distributions of *Phytolacca.* We estimated ancestral ranges under the BAYAREALIKE + j model, which was the best-fit model (Matzke, 2012), using BioGeoBEARS as implemented in Reconstruct Ancestral State in Phylogenies (RASP) version 4.0 (Yu et al., 2020). The dispersal probabilities among regions were set to three categories: 0.01 for well-separated areas, 0.5 for moderately separated areas, and 1.0 for well-connected areas.

## Results

### Chloroplast Genome Features of *Phytolacca*

The *Phytolacca* chloroplast genomes had a quadripartite structure typical of most angiosperm species, including large single copy (LSC) and small single copy (SSC) regions separated by two inverted repeat (IRa and IRb) regions (Supplementary Figure 1), and the sequence lengths and structures were very similar (Table 1 and Supplementary Figure 1). The chloroplast genome size ranged from 155,082 bp (*P. americana* SY851192) to 156,734 bp (*P. acinosa* SY851196), the LSC ranged from 85,272 to 86,425 bp, and the SSC varied between 18,331 and 18,640 bp. The GC content of the chloroplast genome sequences was 36.8–36.9%. The *Phytolacca* chloroplast genome encodes 114 unique genes, including 79 protein-coding genes, 31 transfer RNA (tRNA) genes, and four ribosomal RNA (rRNA) genes. The gene order was highly conserved, and 18 genes had introns in the *Phytolacca* chloroplast genome. There were not differences in the boundaries of LSC/SSC/IR in the *Phytolacca* chloroplast genomes.

**TABLE 1:** Chloroplast genome features of *Phytolacca* chloroplast genomes.

Species	Voucher	LSC	IR	SSC	Total	GC%	Number of genes	Protein coding genes	tRNA	rRNA
*P. acinosa*	SY851188	86,375	25,989	18,332	156,685	36.8	114	79	31	4
*P. acinosa*	SY851196	86,425	25,989	18,331	156,734	36.8	114	79	31	4
*P. americana*	SY851192	85,272	25,585	18,640	155,082	36.9	114	79	31	4
*P. americana*	SY851193	85,282	25,585	18,640	155,092	36.9	114	79	31	4
*P. americana*	SY851194	85,282	25,585	18,640	155,092	36.9	114	79	31	4
*P. americana*	SY851197	85,282	25,585	18,640	155,092	36.9	114	79	31	4
*P. americana*	SY851198	85,282	25,585	18,640	155,092	36.9	114	79	31	4
*P. americana*	SY851199	85,282	25,585	18,640	155,092	36.9	114	79	31	4
*P. dioica*	SY851184	85,659	25,575	18,556	155,365	36.8	114	79	31	4
*P. dioica*	SY851185	85,657	25,575	18,557	155,364	36.8	114	79	31	4
*P. icosandra*	SY851200	85,945	25,555	18,550	155,605	36.8	114	79	31	4
*P. japonica*	SY851190	86,256	25,989	18,357	156,591	36.8	114	79	31	4
*P. latbenia*	SY851186	86,276	25,997	18,363	156,633	36.8	114	79	31	4
*P. polyandra*	SY851189	86,310	25,989	18,340	156,628	36.8	114	79	31	4
*P. rivinoides*	SY851187	85,964	25,538	18,536	155,576	36.8	114	79	31	4
*P. thyrsiflora*	SY851191	86,050	25,573	18,593	155,789	36.8	114	79	31	4
										

### *Phytolacca* Chloroplast Genome Variation

The 18 entire *Phytolacca* chloroplast genomes had an aligned length of 159,444 bp (Table 2), including 2,925 variable sites (1.83%) and 2,616 parsimony-informative sites (1.64%). The overall nucleotide diversity (π) was 0.00614; moreover, each region of the chloroplast genome revealed different sequence divergences; IR exhibited the lowest π value of 0.00136, and SSC had the highest π value of 0.01298. The genetic p-distance of *Phytolacca* species is shown in Supplementary Figure 2. The mean genetic distance was 0.0062, the lowest divergence (0.0002) was between *P. acinosa* and *P. insularis*, and the largest sequence divergence (0.0113) was between *P. dioica* and *P. japonica*.

**TABLE 2:** Analyses of variable sites in chloroplast genomes of *Phytolacca*.

Regions	Length	Variable sites		Information sites		Nucleotide diversity
			
		Numbers	%	Numbers	%	
LSC	82,739	2,014	2.43	1,805	2.18	0.00753
SSC	19,021	677	3.56	605	3.18	0.01298
IR	25,679	117	0.46	103	0.40	0.00136
Complete cp genome	159,444	2,925	1.83	2,616	1.64	0.00614
						

Using the slide window method, π values ranged from 0 to 0.03351 in a 600 bp window size. In total, nine peaks with π values > 0.02 were identified in the *Phytolacca* chloroplast genome (Supplementary Figure 3). These regions included *psbI-trnS-trnG*, *psbM-trnD*, *trnT-trnL*, *ndhC-trnV*, *psbE-petL*, *ndhF*, *ndhF-rpl32-trnL*, *ndhH-rps15*, and *ycf1*. These regions include seven intergenic regions and two coding regions (*ndhF* and *ycf1*). Five intergenic regions were located in the LSC region, and four regions were located in the SSC regions.

We compared nine hypervariable markers and three universal DNA barcodes (*rbcL, matK*, and *trnH-psbA*) and tested the variability of these markers. The variable information is shown in Table 3. The nine hypervariable markers ranged from 612 (*ndhF*) to 3,097 bp (*ndhF-rpl32-trnL*) in length. *ndhF-rpl32-trnL* had the greatest number of variable sites (176 sites), followed by *ndhC-trnV* (85 sites) and *psbI-trnS-trnG* (61 sites). The three universal DNA barcodes had 23, 32, and 27 variable sites, respectively. According to the π values, most of the hypervariable markers were more variable than the three universal DNA barcode markers.

**TABLE 3:** Nine hypervariable regions of chloroplast genomes of *Phytolacca*.

Markers	Length	Variable sites		Parsimony-informative sites		Nucleotide diversity (π)
			
		Numbers	%	Numbers	%	
*rbcL*	1,449	23	1.59	22	1.52	0.00533
*matK*	1,512	32	2.12	30	1.98	0.00804
*trnH-psbA*	506	27	5.34	23	4.55	0.01675
*psbI-trnS-trnG*	934	61	6.53	52	5.57	0.02206
*psbM-trnD*	805	47	5.84	43	5.34	0.01997
*trnT-trnL*	990	45	4.55	39	3.94	0.01837
*ndhC-trnV*	1,501	85	5.66	77	5.13	0.02002
*psbE-petL*	793	37	4.67	37	4.67	0.01863
*ndhF*	612	32	5.23	31	5.07	0.02112
*ndhF-rpl32-trnL*	3,097	176	5.68	163	5.26	0.02318
*ndhH-rps15*	712	38	5.34	35	4.92	0.02064
*ycf1*	906	49	5.41	42	4.64	0.0181

A total of 57–76 SSRs were found in the *Phytolacca* chloroplast genomes. Mono-, di-, tri-, tetra-, penta-, and hexanucleotide SSRs were identified (Supplementary Figure 4). The majority of SSRs (68.06%) were mononucleotide repeats in all *Phytolacca* species, followed by tetranucleotide repeats. Most mononucleotide repeats were composed of A/T with minimal G/C.

We identified thirteen small inversions in the *Phytolacca* chloroplast genome (Table 4). All inversions and their inverted repeating flanking sequences formed stem–loop structures. The inversion length was 2–29 bp, and the flanking repeats ranged from 4 to 27 bp. The longest inversion occurred in the *petA-psbJ* region. Eight small inversions were located in the LSC region, one in the IR region, and four in the SSC region. Most inversions were located in the non-coding region, including ten in intergenic regions and one in the intron region (*ndhB*). Two inversions occurred in coding regions (*ndhD* and *ycf1*).

**TABLE 4:** The size and locations of small inversions in *Phytolacca* chloroplast genomes.

Region	Position	Location	Length of loop (bp)	Length of stem (bp)
LSC	*rpoB-trnC*	spacer	4	9
LSC	*trnC-petN*	spacer	3	8
LSC	*petN-psbM*	spacer	10	18
LSC	*ndhC-trnV*	spacer	3	20
LSC	*trnM-atpE*	spacer	7	20
LSC	*trnM-atpE*	spacer	10	27
LSC	*accD-psaI*	spacer	7	12
LSC	*petA-psbJ*	spacer	29	22
IR	*ndhB*	intron	2	4
SSC	*rpl32-trnL*	spacer	2	24
SSC	*trnL-ndhD*	spacer	10	16
SSC	*ndhD*	exon	4	23
SSC	*ycf1*	exon	3	9

### Phylogenetic Relationships

The 19cpg dataset matrix contained 19 Phytolaccaceae *s.s.* chloroplast genome samples, of which 159,748 were aligned nucleotide sites. The second data matrix, 83g48s, contained 79 protein-coding genes and four rRNA genes from 48 Phytolaccaceae *s.l.* and its ally samples. This dataset contained 68,638 nucleotide sites, including 11,194 variable sites and 7,634 parsimony-informative sites. The 3g22s dataset contained three markers (nrDNA, *rbcL* and *matK*), including 22 *Phytolacca* species and three outgroups. This dataset matrix included 5,752 nucleotide sites, of which 538 were variable sites.

**FIGURE 1:**
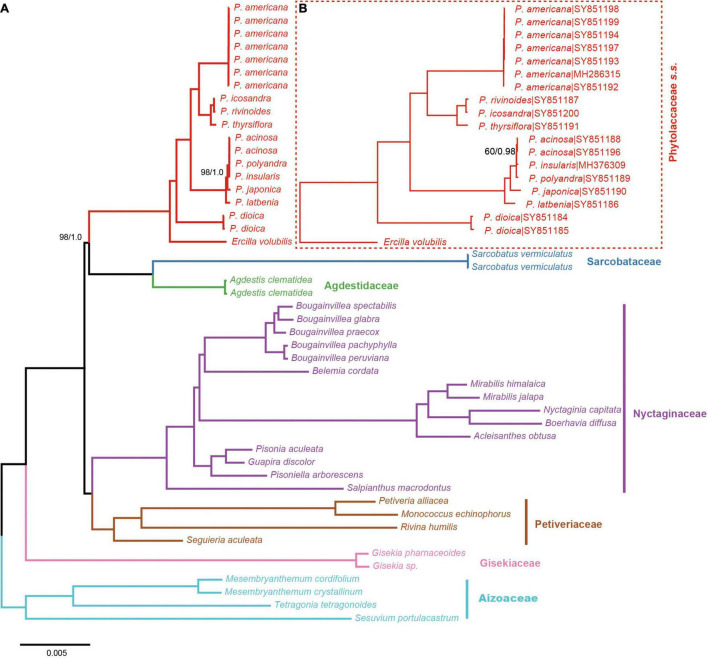
Phylogenetic trees of Phytolaccaceae *s.l.* and its allies obtained from an ML and BI analysis of the complete chloroplast genome. **(A)** ML tree with a strict hierarchical clustering partitioning scheme using the 83g48s dataset. **(B)** ML tree with GTR + G model using the 19cpg dataset. ML bootstrap support value/Bayesian posterior probability presented at each node. Node support values of ML = 100/BI = 1.0 were not shown.

All relationships among the sampled major clades of the Phytolaccaceae *s.l.* and its allies were well resolved and strongly supported using the 83g48s dataset (Figure 1). Gisekiaceae was sister to everything except Aizoaceae. Nyctaginaceae and Petiveriaceae formed a clade (BS = 100/PP = 1). Sarcobataceae and Agdestidaceae formed a clade which was sister to Phytolaccaceae *s.s.* (BS = 98/PP = 1).

The results from the 19 cpg and 83g48s datasets were similar to the phylogenetic relationships of *Phytolacca* species (Figure 1). All *Phytolacca* species formed a monophyletic group (BS = 100/PP = 1) and were sister to *Ercilla volubilis* within the Phytolaccaceae *s.s.* according to the 83g48s dataset (Figure 1). *P. dioica* was the firstly diverged species. Five species (*P. acinosa*, *P. insularis*, *P. japonica*, *P. latbenia*, and *P. polyandra*) formed a monophyletic group with high support value (BS = 100/PP = 1). All individuals of *P. americana* were in a clade and sister to the group containing three species (*P. rivinoides, P. icosandra*, and *P. thyrsiflora*) with high support values (BS = 100/PP = 1).

**FIGURE 2:**
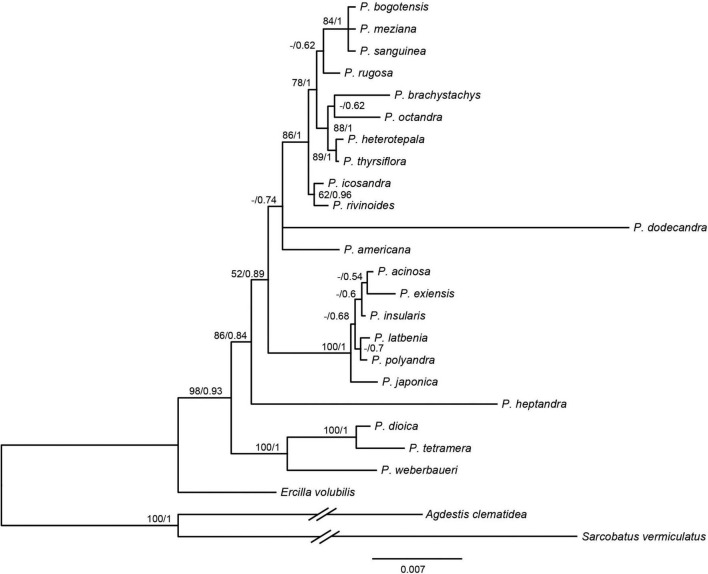
Phylogenetic trees of *Phytolacca* based on the 3g22s dataset. ML bootstrap support values/Bayesian posterior probabilities were presented at each node.

Based on the 3g22s dataset, the phylogenetic relationships within *Phytolacca* have low supports in some nodes (Figure 2). Three species of *P. dioica, P. tetramera* and *P. weberbaueri* formed a clade with high support values (BS = 100/PP = 1) and *P. weberbaueri* was sister to the other two species. This clade was the firstly diverged group of *Phytolacca* and was sister to the remaining species. *Phytolacca heptandra* was the secondly diverged group, however, this relationship was weakly supported (BS = 86/PP = 0.84). The six species of *P. acinosa*, *P. exiensis*, *P. insularis*, *P. japonica*, *P. latbenia*, and *P. polyandra* formed a highly supported clade (BS = 100/PP = 1). The phylogenetic position of *P. dodecandra* and *P. americana* were uncertain. The remaining ten species formed a clade with moderate supported (BS = 86/PP = 1).

### Divergence Times

**FIGURE 3:**
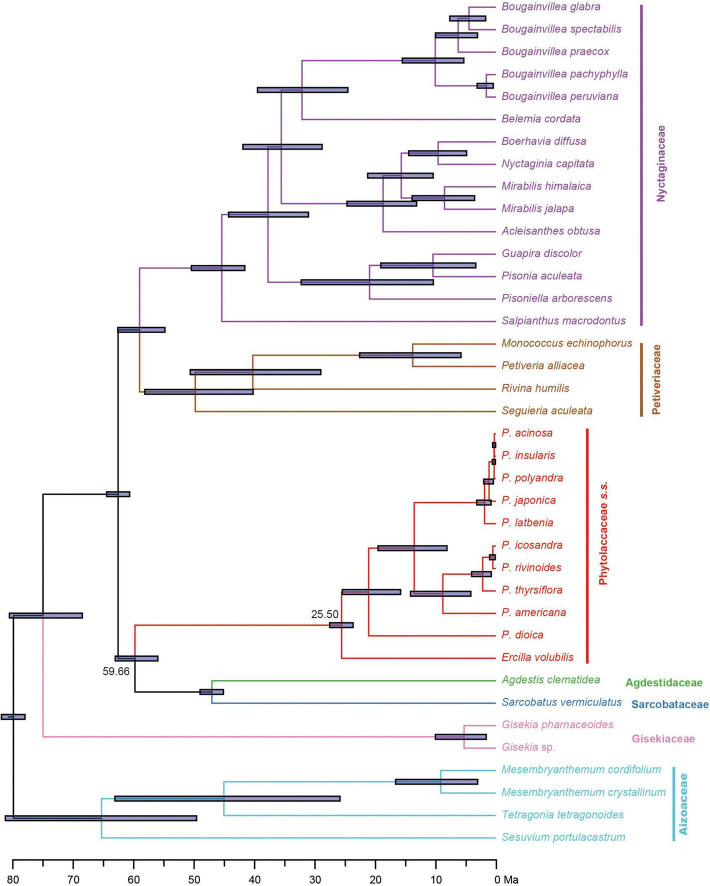
Divergence times of Phytolaccaceae *s.l.* and its allies obtained from BEAST analysis based on the 83g48s dataset. The mean divergence time of the nodes is shown next to the nodes, while the blue bars correspond to the 95% highest posterior density (HPD). Black circles indicate the five calibration points.

Divergence time estimates based on the 83g48s dataset suggested that the stem and crown of Phytolaccaceae *s.s.* were 59.66 Ma [95% highest posterior densities (HPD): 55.88–62.91 Ma] in the Paleocene and 25.50 Ma (95% HPD: 23.59–27.47 Ma) during the later Oligocene (Figure 3). Using the 3g22s dataset, the species-level divergence times were estimated using three priors (Supplementary Figure 5). The crown age of *Phytolacca* was 20.30 Ma (95% HPD: 15.79–24.60 Ma) in the Miocene. Most deep nodes diverged in the Miocene, and all extant *Phytolacca* species diverged in the Pliocene.

### Historic Biogeography of *Phytolacca*

**FIGURE 4:**
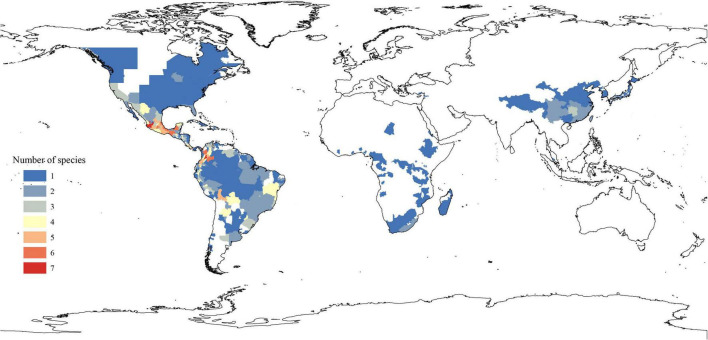
Spatial patterns of *Phytolacca* species diversity.

The extant *Phytolacca* species has a clear pattern of intercontinentally disjunct distribution (Figure 4). There are four native areas: Central and South America, North America, Asia, and Africa. The greatest diversity is found in Central America, including Mexico, Costa Rica, and Colombia. The next highest diversity is found in South America. In contrast, diversity is slightly lower in North America and Africa.

**FIGURE 5:**
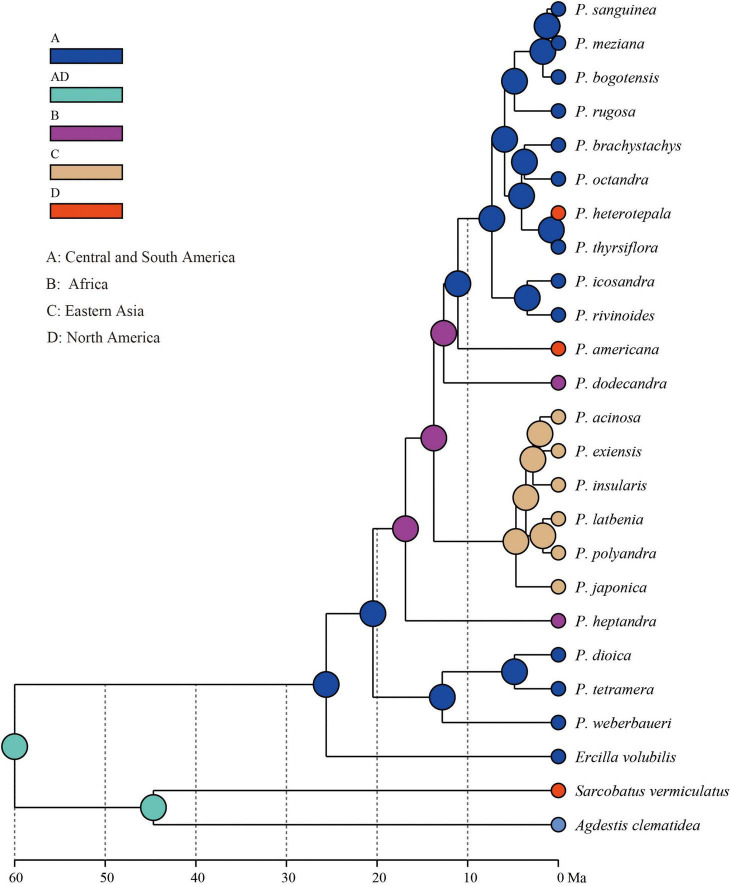
Ancestral area reconstructions within *Phytolacca* using the 3g22s dataset.

The ancestral distributions inferred from BioGeoBEARS for internal nodes in *Phytolacca* are shown in Figure 5. The results suggested Central and South America (A) as the ancestral area for *Phytolacca.* Five dispersal events were identified within *Phytolacca* (Figure 5). The first was the ancestor of *Phytolacca* dispersed to Africa (B), and the second dispersal event was from Africa (B) to Eastern Asia (C) and gave rise to Eastern Asia clade. The third dispersal event from Africa (B) to Central and South America (A). There were two dispersal events from Central and South America (A) to North America (D), which gave rise to the species *P. americana* and *P. heterotepala*.

## Discussion

### Phylogeny of *Phytolacca*

The molecular phylogenetic results using the ITS (Ali et al., 2015), several genes (Figure 2) and chloroplast genome data (Figure 1) concluded that the relationships among the species within the infrageneric are inconsistent with the generic classification based on morphology. Our phylogenetic tree is mostly consistent with Ali et al. (2015); Figure 2). The relationships of the deep nodes were not inconsistent. Ali et al. (2015) supported *P. heptandra* was the firstly diverged group, while our result supported a clade with three species of *P. dioica, P. tetramera* and *P. weberbaueri* (Figure 2). The three species are supported by morphological characteristics, such as carpels with more or less united, diecious flowers. These three species are under the subgenus *Pircuniopsis* Sect. *Pircuniopsis* (Nowicke, 1968) (Supplementary Figure 6). *Phytolacca heptandra* is in the subgenus *Pircunia* Sect. *Pircunia* with *P. esculenta*, *P. acinosa*, *P. latbenia* and *P. cyclopetala* according to Walter’s (1909) infrageneric classification. The 3g22s dataset did not resolve the position of *P. heptandra* due to less information in sequences (Figure 2), and unfortunately, we did not possess the chloroplast genome sequence of this species, which was narrowly distributed in South Africa (Nowicke, 1968).

The remaining *Phytolacca* species [clade III in Ali et al. (2015); Figure 2] mainly belonged to the subgenus *Phytolacca* Sect. *Phytolacca* (Nowicke, 1968). The monophyly of these species was supported with 100% ML bootstrap support and 1 Bayesian posterior probability by 19cpg and 83g48s datasets (Figure 1). However, the relationships in this clade were unclear using the three gene dataset (Figure 2).

A well supported clade included six species in eastern Asia. This group of very robust herbs with conspicuously free-carpelled ovaries is treated as an “aggregate species.” The taxonomy of this group was controversial, and the number of species ranged from one to seven (Nowicke, 1968; Lu and Kai, 2003). Two new species (*P. exiensis* and *P. yunnanensis*) were published based on morphological characteristics or the molecular data in recent years (Xie et al., 2017; Li et al., 2020). *P. exiensis* displays carpers connate and flowers bisexus and flowers green at the early stage, morphological characteristics suggest that this species is closely related to *P. dodecandra* (from the Africa and Madagascar) and molecular data suggests that this species is closely related to *P. acinose* (Xie et al., 2017). *Phytolacca yunnanensis* is also similar to *P. exiensis* by the number of carpels, but can be easily distinguished from the latter in having free carpels (Li et al., 2020). The phylogenetic trees inferred from the chloroplast genome data and the multiple genes data showed that some species were less divergent (Figure 1 and Supplementary Figure 5). Further work based on expanded sampling is needed to test the phylogenetic relationships and perform taxonomy using more molecular data.

*Phytolacca americana* was included in the subgenus *Phytolacca* Sect. *Phytolacca* (Nowicke, 1968). The phylogenetic relationship was consistent with a recent study based on ITS (Ali et al., 2015). These results suggested that the phylogenetic position of *P. americana* was unclear and was more different from other species of the subgenus *Phytolacca* Sect. *Phytolacca*. *Phytolacca dodecandra* was included in the section (subgenus *Pircunia* Sect. *Pircunioides*) according to the carpel being completely free and the diecious flowers (Nowicke, 1968) (Supplementary Figure 6). The 3g22s dataset showed *P. dodecandra* had longer branch length and the relationship was not resolved (Figure 2).

The largest clade of *Phytolacca* with moderate support values included ten species, belonging to the subgenus *Phytolacca* Sect. *Phytolacca* (*P. brachystachys*, *P. bogotensis*, *P. heterotepala*, *P. icosandra*, *P. meziana*, *P. octandra*, *P. rivinoides*, and *P. thyrsiflora*) and subgenus *Pircuniopsis* Sect. *Pircuniophorum* (*P. rugosa* and *P. sanguinea*) with hermaphroditic flowers. However, the phylogenetic relationships of these species were unresolved using three genes (Figure 2).

In this study, we used multiple datasets to infer the phylogenetic relationships of *Phytolacca*, and our results provided new insights for resolving the classification of *Phytolacca.* Compared to the three markers datasets, the 19cpg/83g48s datasets had higher resolution, however, these two datasets both had insufficient sampling issues that they did not cover all of the species. The 3g22s dataset included most of living *Phytolacca* species, whereas, the inferred phylogenetic tree had lower support values in many nodes. The whole chloroplast genome sequences contained more variable loci to resolve a better supporting phylogeny, indicating that more sampling of both species and chloroplast genomes would be needed in future for understanding the evolving history of *Phytolacca*.

### Divergence Time and Biogeography of *Phytolacca*

The origin time of *Phytolacca* was estimated to have begun at 25.50 Ma in the later Oligocene (Supplementary Figure 5) and began diversification from the Miocene. The majority of extant plant species diversified from the Miocene owing to the global climate transition from warm and humid conditions to seasonal climates (Kürschner et al., 2008). Our BioGeoBEARS analysis inferred the ancestral area of *Phytolacca* in Central and South America. We identified five dispersal events to be *Phytolacca* (Figure 5). Using the specimen records, we drew the distribution pattern of *Phytolacca* (Figure 4). The extant species presents the intercontinentally disjunct distribution, and central America is the center of distribution. *Phytolacca* species, for example, pokeweeds (*P. americana*), have the ability to become established quickly far from parental plants upon disturbance of soil and vegetation (Rogers, 1985). Pokeweeds were now widespread in China and had become an invasive plant since it was first introduced in the 1930s (Yang et al., 2010). More studies draw attention to their seeds. Often, fleshy-fruited invasive plants easily form seed dispersal mutualisms with resident fauna, particularly birds (Cruz et al., 2013). Pokeweed seeds remain viable in fecal deposits from birds, which undoubtedly are the primary agents of dispersal (McDonnell et al., 1984), and *Pycnonotus sinensis* and *Urocissa erythrorhyncha* are the most frequent dispersers in China (Li et al., 2017).

## Data Availability Statement

The datasets presented in this study can be found in online repositories. The names of the repository/repositories and accession number(s) can be found in the article/Supplementary Material.

## Author Contributions

YS, FJ, and SZ designed the experiment and drafted and made revisions to the manuscript. JS collected the samples and performed the experiment. YS and CW analyzed the data. NX and SZ contributed to the reagents and analysis tools. All authors have read and agreed to the published version of the manuscript.

## Conflict of Interest

The authors declare that the research was conducted in the absence of any commercial or financial relationships that could be construed as a potential conflict of interest.

## Publisher’s Note

All claims expressed in this article are solely those of the authors and do not necessarily represent those of their affiliated organizations, or those of the publisher, the editors and the reviewers. Any product that may be evaluated in this article, or claim that may be made by its manufacturer, is not guaranteed or endorsed by the publisher.

## Footnotes

1
http://pgrc.ipk-gatersleben.de/misa/misa.html

2
https://www.gbif.org/

3
http://www.nsii.org.cn/

4
http://www.plantsoftheworldonline.org/

## Supplementary Material

Supplementary Figure 1 Map of the chloroplast genome of the genus *Phytolacca.*

Supplementary Figure 2 Pairwise genetic distances among *Phytolacca* species.

Supplementary Figure 3 Hypervariable regions within the chloroplast genome of *Phytolacca* species using sliding window analysis. Window lengths: 600 bp; step size: 50 bp. Nine regions with the highest Pi values were marked out. LSC, large single-copy region, IR, inverted repeat region, SSC, small single-copy region. *x*-axis: position of the midpoint of a window; *y*-axis: nucleotide diversity of each window.

Supplementary Figure 4 Frequency of SSRs in the *Phytolacca* chloroplast genomes. **(A)** The number of SSRs detected in the different *Phytolacca* species; **(B)** The number of SSR types in different *Phytolacca* species.

Supplementary Figure 5 Divergence times of *Phytolacca* obtained from BEAST analysis based on the 3g22s dataset. The mean divergence time of the nodes is shown next to the nodes, while the blue bars correspond to the 95% highest posterior density (HPD).

Supplementary Figure 6 Phylogenetic trees of *Phytolacca* based on the 3g22s dataset. ML bootstrap support values/Bayesian posterior probabilities were presented at each node. The infrageneric classification of the genus *Phytolacca* by Nowicke (1968) was mapped in the tree. The species which were not marked with the subgenus/section, were recently described or not accepted by Nowicke (1968).

## References

[B1] Ali M. A. Lee J. Kim S.-Y. Park S.-H. Al-Hemaid F. M. (2015). Molecular phylogenetic analyses of internal transcribed spacer (ITS) sequences of nuclear ribosomal DNA indicate monophyly of the genus Phytolacca L.(*Phytolaccaceae*). *Bangladesh J. Plant Taxon.* 22 1–8.

[B2] Bentley K. E. Berryman K. R. Hopper M. Hoffberg S. L. Myhre K. E. Iwao K. (2015). Eleven microsatellites in an emerging invader, Phytolacca americana (*Phytolaccaceae*), from its native and introduced ranges. *Appl. Plant Sci.* 3:1500002. 10.3732/apps.1500002 25798346 PMC4356323

[B3] Bolger A. M. Lohse M. Usadel B. (2014). Trimmomatic: a flexible trimmer for Illumina sequence data. *Bioinformatics* 30 2114–2120. 10.1093/bioinformatics/btu170 24695404 PMC4103590

[B4] Borak Martan V. Šoštarić R. (2016). Phytolacca acinosa Roxb.(*Phytolaccaceae*), a new alien species in the Croatian flora. *Acta Bot. Croat.* 75 206–209.

[B5] Bouckaert R. Heled J. Kuhnert D. Vaughan T. Wu C. H. Xie D. (2014). BEAST 2: a software platform for Bayesian evolutionary analysis. *PLoS Comput. Biol.* 10:e1003537. 10.1371/journal.pcbi.1003537 24722319 PMC3985171

[B6] Caulkins D. B. Wyatt R. (1990). Variation and Taxonomy of Phytolacca americana and P. rigida in the Southeastern United States. *Bull. Torrey Bot. Club* 117 357–367.

[B7] Choe S. Jeong S. Jang M. Yeom H. Moon S. Kang M. (2020). Identification of phytolaccosides in biological samples from pokeweed intoxication patients using liquid chromatography-tandem mass spectrometry. *J. Chromatogr. B* 1149:122123. 10.1016/j.jchromb.2020.122123 32480320

[B8] Cruz J. C. Ramos J. A. Da Silva L. P. Tenreiro P. Q. Heleno R. H. (2013). Seed dispersal networks in an urban novel ecosystem. *Eur. J. For. Res.* 132 887–897.

[B9] Dong W. Liu Y. Li E. Xu C. Sun J. Li W. (2022a). Phylogenomics and biogeography of Catalpa (Bignoniaceae) reveal incomplete lineage sorting and three dispersal events. *Mol. Phylogenet. Evol.* 166:107330. 10.1016/j.ympev.2021.107330 34687844

[B10] Dong W. Sun J. Liu Y. Xu C. Wang Y. Suo Z. (2022b). Phylogenomic relationships and species identification of the olive genus *Olea* (*Oleaceae*). *J. Syst. Evol.* 10.1111/jse.12802

[B11] Dong W. Liu Y. Xu C. Gao Y. Yuan Q. Suo Z. (2021a). Chloroplast phylogenomic insights into the evolution of *Distylium* (*Hamamelidaceae*). *BMC Genomics* 22:293. 10.1186/s12864-021-07590-6 33888057 PMC8060999

[B12] Dong W. Xu C. Liu Y. Shi J. Li W. Suo Z. (2021b). Chloroplast phylogenomics and divergence times of *Lagerstroemia* (*Lythraceae*). *BMC Genomics* 22:434. 10.1186/s12864-021-07769-x 34107868 PMC8191006

[B13] Fassett N. C. Sauer J. D. (1950). Studies of Variation in the Weed Genus phytolacca. I. Hybridizing Species in Northeastern Colombia. *Evolution* 4 332–339.

[B14] Greiner S. Lehwark P. Bock R. (2019). OrganellarGenomeDRAW (OGDRAW) version 1.3.1: expanded toolkit for the graphical visualization of organellar genomes. *Nucleic Acids Res.* 47 W59–W64. 10.1093/nar/gkz238 30949694 PMC6602502

[B15] Huang D. I. Cronk Q. C. B. (2015). Plann: a command-line application for annotating plastome sequences. *Appl. Plant Sci.* 3:1500026. 10.3732/apps.1500026 26312193 PMC4542940

[B16] Jin J.-J. Yu W.-B. Yang J.-B. Song Y. Depamphilis C. W. Yi T.-S. (2020). GetOrganelle: a fast and versatile toolkit for accurate de novo assembly of organelle genomes. *Genome Biol.* 21:241. 10.1186/s13059-020-02154-5 32912315 PMC7488116

[B17] Kalyaanamoorthy S. Minh B. Q. Wong T. K. F. Von Haeseler A. Jermiin L. S. (2017). ModelFinder: fast model selection for accurate phylogenetic estimates. *Nat. Methods* 14 587–589. 10.1038/nmeth.4285 28481363 PMC5453245

[B18] Katoh K. Standley D. M. (2013). MAFFT multiple sequence alignment software version 7: improvements in performance and usability. *Mol. Biol. Evol.* 30 772–780. 10.1093/molbev/mst010 23329690 PMC3603318

[B19] Kozlov A. M. Darriba D. Flouri T. Morel B. Stamatakis A. (2019). RAxML-NG: a fast, scalable and user-friendly tool for maximum likelihood phylogenetic inference. *Bioinformatics* 35 4453–4455. 10.1093/bioinformatics/btz305 31070718 PMC6821337

[B20] Kumar S. Stecher G. Tamura K. (2016). MEGA7: molecular evolutionary genetics analysis version 7.0 for bigger datasets. *Mol. Biol. Evol.* 33 1870–1874. 10.1093/molbev/msw054 27004904 PMC8210823

[B21] Kürschner W. M. Kvaček Z. Dilcher D. L. (2008). The impact of Miocene atmospheric carbon dioxide fluctuations on climate and the evolution of terrestrial ecosystems. *Proc. Natl. Acad. Sci. U. S. A.* 105:449. 10.1073/pnas.0708588105 18174330 PMC2206556

[B22] Lee J. Kim S. Y. Park S.-H. Ali M. (2013). Molecular phylogenetic relationships among members of the family *Phytolaccaceae* sensu lato inferred from internal transcribed spacer sequences of nuclear ribosomal DNA. *Genet. Mol. Res.* 12 4515–4525. 10.4238/2013.February.28.15 23479160

[B23] Li J. Wang S. Jing Y. Wang L. Zhou S. (2013). A modified CTAB protocol for plant DNA extraction. *Chin. Bull. Bot.* 48 72–78.

[B24] Li N. Yang W. Fang S. Li X. Liu Z. Leng X. (2017). Dispersal of invasive Phytolacca americana seeds by birds in an urban garden in China. *Integr. Zool.* 12 26–31. 10.1111/1749-4877.12214 27265341

[B25] Li X. Zhou W.-B. Guo J.-C. Yin X.-M. (2020). Phytolacca yunnanensis (*Phytolaccaceae*), a new species from China with distinctive inflorescence characteristics. *Phytotaxa* 446 49–54.

[B26] Lu D. Kai L. (2003). “Phytolacca,” in *Flora of China*, eds Wu Z. Y. Raven P. H. Hong D. Y. (Beijing, St Louis: Science Press), 435–436.

[B27] Matzke N. J. (2012). Founder-event speciation in BioGeoBEARS package dramatically improves likelihoods and alters parameter inference in Dispersal-Extinction-Cladogenesis (DEC) analyses. *Front. Biogeogr.* 4:210.

[B28] McDonnell M. J. Stiles E. W. Cheplick G. P. Armesto J. J. (1984). Bird-dispersal of Phytolacca americana L. and the influence of fruit removal on subsequent fruit development. *Am. J. Bot.* 71 895–901.

[B29] Monkiedje A. Anderson A. C. Englande A. J. (1991). Acute toxicity of Phytolacca dodecandra (Endod-S) and Niclosamide to snails, Schistosoma mansoni cercaria, Tilapia fish, and soil microorganisms. *Environ. Toxicol. Water Qual.* 6 405–413.

[B30] Nowicke J. W. (1968). Palynotaxonomic Study of the *Phytolaccaceae*. *Ann. Mo. Bot. Gard.* 55 294–364.

[B31] Rambaut A. Drummond A. Suchard M. J. T. (2014). *Tracer v1.6.* Available online at: http://beast.bio.ed.ac.uk

[B32] Rogers G. K. (1985). The genera of *Phytolaccaceae* in the Southeastern United States. *J. Arnold Arbor.* 66 1–37.

[B33] Rohwer J. G. (1993). “Phytolaccaceae,” in *Flowering Plants ⋅ Dicotyledons: Magnoliid, Hamamelid and Caryophyllid Families*, eds Kubitzki K. Rohwer J. G. Bittrich V. (Berlin, Heidelberg: Springer Berlin Heidelberg), 506–515.

[B34] Ronquist F. Teslenko M. Van Der Mark P. Ayres D. L. Darling A. Hohna S. (2012). MrBayes 3.2: efficient Bayesian phylogenetic inference and model choice across a large model space. *Syst. Biol.* 61 539–542. 10.1093/sysbio/sys029 22357727 PMC3329765

[B35] Rozas J. Ferrer-Mata A. Sanchez-Delbarrio J. C. Guirao-Rico S. Librado P. Ramos-Onsins S. E. (2017). DnaSP 6: DNA sequence polymorphism analysis of large data sets. *Mol. Biol. Evol.* 34 3299–3302. 10.1093/molbev/msx248 29029172

[B36] Schäferhoff B. MÜller K. F. Borsch T. (2009). Caryophyllales phylogenetics: disentangling *Phytolaccaceae* and Molluginaceae and description of Microteaceae as a new isolated family. *Willdenowia* 39 209–228.

[B37] The Angiosperm Phylogeny Group (2016). An update of the Angiosperm Phylogeny Group classification for the orders and families of flowering plants: APG IV. *Bot. J. Linn. Soc.* 181 1–20.

[B38] Vanvinckenroye P. Smets E. F. (1997). A Study of Floral Morphological Diversity in Phytolacca (*Phytolaccaceae*) Based on Early Floral Ontogeny. *Int. J. Plant Sci.* 158 57–72.

[B39] Walter H. (1909). Phytolaccaceae. *Pflanzenr* 83 1–154.

[B40] Willis J. C. (1966). *A Dictionary of the Flowering Plants and Ferns.* Cambridge: University Press.

[B41] Xiao Y. Li Y. Shi Y. Li Z. Zhang X. Liu T. (2022). Combined toxicity of zinc oxide nanoparticles and cadmium inducing root damage in Phytolacca americana L. *Sci. Total Environ.* 806:151211. 10.1016/j.scitotenv.2021.151211 34715219

[B42] Xie D. Qian D. Zhang M. Wang Y. Wu Y. Huang L. (2017). Phytolacca exiensis, a new species of *Phytolaccaceae* from west of Hubei province, China. *Phytotaxa* 331 224–232.

[B43] Yang B. Zhuoga Y. Pan X. Xu H. Li B. (2010). Alien terrestrial herbs in China: diversity and ecological insights. *Biodivers. Sci.* 18 660–666.

[B44] Yao G. Jin J.-J. Li H.-T. Yang J.-B. Mandala V. S. Croley M. (2019). Plastid phylogenomic insights into the evolution of Caryophyllales. *Mol. Phylogenet. Evol.* 134 74–86. 10.1016/j.ympev.2018.12.023 30735725

[B45] Yu Y. Blair C. He X. (2020). RASP 4: ancestral State Reconstruction Tool for Multiple Genes and Characters. *Mol. Biol. Evol.* 37 604–606. 10.1093/molbev/msz257 31670774

[B46] Zetter R. Hofmann C.-C. Draxler I. Durango De Cabrera J. Del Mvergel M. Vervoorst F. (1999). A rich middle Eocene microflora at Arroyo de los Mineros, near Cañadón Beta, NE Tierra del Fuego province, Argentina. *Abh. Geol. Bundesanst.* 56 439–460.

