# Supplementary material for: Phylogenomics Reveals the Evolutionary History of Phytolacca (Phytolaccaceae)
Source: Front Plant Sci. 2022 Jun 10;13:844918. doi: 10.3389/fpls.2022.844918 (PMC9226614; doi:10.3389/fpls.2022.844918)
Supplement: Supplementary file 1 [file Table_1.DOCX]

Table S1. Taxa included in this study with locality, voucher, and GenBank accession numbers.

| Taxon | Locality | Preservation | Accession number of plastome | Accession number of nrDNA | Voucher |
| --- | --- | --- | --- | --- | --- |
| *Phytolacca dioica* | Morretes, Brazil | DNA bank | OM403703 | OL824846 | SY851184 |
| *Phytolacca dioica* | Central, Paraguay | DNA bank | OM403712 | OL824847 | SY851185 |
| *Phytolacca latbenia* | Jingdong, Yunnan, China | Silicagel | OM403704 | OL824848 | SY851186 |
| *Phytolacca rivinoides* | Municipio de Wiwili. Reserva Cerro Kilambe, Nicaragua | DNA bank | OM403718 | OL824849 | SY851187 |
| *Phytolacca acinosa* | Ningxian, Shaanxi, China | Silicagel | OM403713 | OL824850 | SY851188 |
| *Phytolacca polyandra* | Wenxian, Gansu, China | Silicagel | OM403705 | OL824851 | SY851189 |
| *Phytolacca japonica* | Taizhong, Taiwan, China | Silicagel | OM403706 | OL824852 | SY851190 |
| *Phytolacca thyrsiflora* | Tijuana, Mexico | DNA bank | OM403714 | OL824853 | SY851191 |
| *Phytolacca americana* | Arnold Arboretum, Boston, America | DNA bank | OM403707 | OL824854 | SY851192 |
| *Phytolacca americana* | Heshan, Hunan, China | Silicagel | OM403708 | OL824855 | SY851193 |
| *Phytolacca americana* | Fuyang, Anhui, Chian | Silicagel | OM403709 | OL824856 | SY851194 |
| *Phytolacca acinosa* | Zhanping, Shaanxi, China | Silicagel | OM403715 | OL824857 | SY851196 |
| *Phytolacca americana* | Sishui, Shangdong, China | Silicagel | OM403710 | OL824858 | SY851197 |
| *Phytolacca americana* | Congyi, Jinagxi, China | Silicagel | OM403716 | OL824859 | SY851198 |
| *Phytolacca americana* | Wanyuan, Sichuan, China | Silicagel | OM403711 | OL824860 | SY851199 |
| *Phytolacca icosandra* | Puntarenas, Costa Rica | DNA bank | OM403717 | OL824861 | SY851200 |
