# Supplementary material for: Phylogenomics Reveals the Evolutionary History of Phytolacca (Phytolaccaceae)
Source: Front Plant Sci. 2022 Jun 10;13:844918. doi: 10.3389/fpls.2022.844918 (PMC9226614; doi:10.3389/fpls.2022.844918)
Supplement: Supplementary file 2 [file Table_2.DOCX]

Table S2. The sequences accession numbers of ITS, *rbcL*, and *matK* downloaded from GenBank.

| Species | ITS | rbcL | matK |
| --- | --- | --- | --- |
| *Phytolacca bogotensis* | KM491868 |  |  |
| *Phytolacca brachystachys* | KM491869 |  |  |
| *Phytolacca dodecandra* | KM491870 |  |  |
| *Phytolacca exiensis* | MG595708 |  |  |
| *Phytolacca heptandra* | KM491871 |  |  |
| *Phytolacca heterotepala* | KM491872 |  |  |
| *Phytolacca insularis* |  | MH376309 | MH376309 |
| *Phytolacca meziana* | KM491873 |  |  |
| *Phytolacca octandra* | KY968825 | MF135319 | MF159384 |
| *Phytolacca rugosa* | KM491877 |  |  |
| *Phytolacca sanguinea* | KM491878 |  |  |
| *Phytolacca tetramera* | KM491879 |  |  |
| *Phytolacca weberbaueri* | KM491881 |  |  |
| *Sarcobatus vermiculatus* | EF079501 | MH286338 | MH286338 |
| *Agdestis clematidea* | JX232581 | MK397910 | MK397910 |
| *Ercilla volubilis* |  | MK397920 | MK397920 |
