# Supplementary material for: Phylogenomics Reveals the Evolutionary History of Phytolacca (Phytolaccaceae)
Source: Front Plant Sci. 2022 Jun 10;13:844918. doi: 10.3389/fpls.2022.844918 (PMC9226614; doi:10.3389/fpls.2022.844918)
Supplement: Supplementary file 3 [file Table_3.DOCX]

Table S3. A list of 30 samples from GenBank for phylogenetic and divergence time analysis.

| Family | Species | GenBank accession numbers |
| --- | --- | --- |
| Agdestidaceae | *Agdestis clematidea* | MH286339 |
| Agdestidaceae | *Agdestis clematidea* | MK397910 |
| Aizoaceae | *Mesembryanthemum cordifolium* | MK397873 |
| Aizoaceae | *Mesembryanthemum crystallinum* | KM016695 |
| Aizoaceae | *Sesuvium portulacastrum* | MK330004 |
| Aizoaceae | *Tetragonia tetragonoides* | MK397903 |
| Gisekiaceae | *Gisekia pharnaceoides* | MK397870 |
| Gisekiaceae | *Gisekia* sp. | MK397921 |
| Nyctaginaceae | *Acleisanthes obtusa* | MH286321 |
| Nyctaginaceae | *Belemia cordata* | MK291267 |
| Nyctaginaceae | *Boerhavia diffusa* | MN711726 |
| Nyctaginaceae | *Bougainvillea glabra* | MW123899 |
| Nyctaginaceae | *Bougainvillea pachyphylla* | MW123902 |
| Nyctaginaceae | *Bougainvillea peruviana* | MW123901 |
| Nyctaginaceae | *Bougainvillea praecox* | MW123900 |
| Nyctaginaceae | *Bougainvillea spectabilis* | MK397858 |
| Nyctaginaceae | *Guapira discolor* | MH286310 |
| Nyctaginaceae | *Mirabilis himalaica* | MN548767 |
| Nyctaginaceae | *Mirabilis jalapa* | MW894644 |
| Nyctaginaceae | *Nyctaginia capitata* | MH286318 |
| Nyctaginaceae | *Pisonia aculeata* | MK397886 |
| Nyctaginaceae | *Pisoniella arborescens* | MH286306 |
| Nyctaginaceae | *Salpianthus macrodontus* | MH286311 |
| Petiveriaceae | *Monococcus echinophorus* | MH286317 |
| Petiveriaceae | *Petiveria alliacea* | MH286334 |
| Petiveriaceae | *Rivina humilis* | MK397894 |
| Petiveriaceae | *Seguieria aculeata* | MH286340 |
| Phytolaccaceae s.s. | *Ercilla volubilis* | MK397920 |
| Sarcobataceae | *Sarcobatus vermiculatus* | MH286338 |
| Sarcobataceae | *Sarcobatus vermiculatus* | MK397895 |
