# Supplementary figures and images for: Phylogenomics Reveals the Evolutionary History of Phytolacca (Phytolaccaceae)
Source: Front Plant Sci. 2022 Jun 10;13:844918. doi: 10.3389/fpls.2022.844918 (PMC9226614; doi:10.3389/fpls.2022.844918)

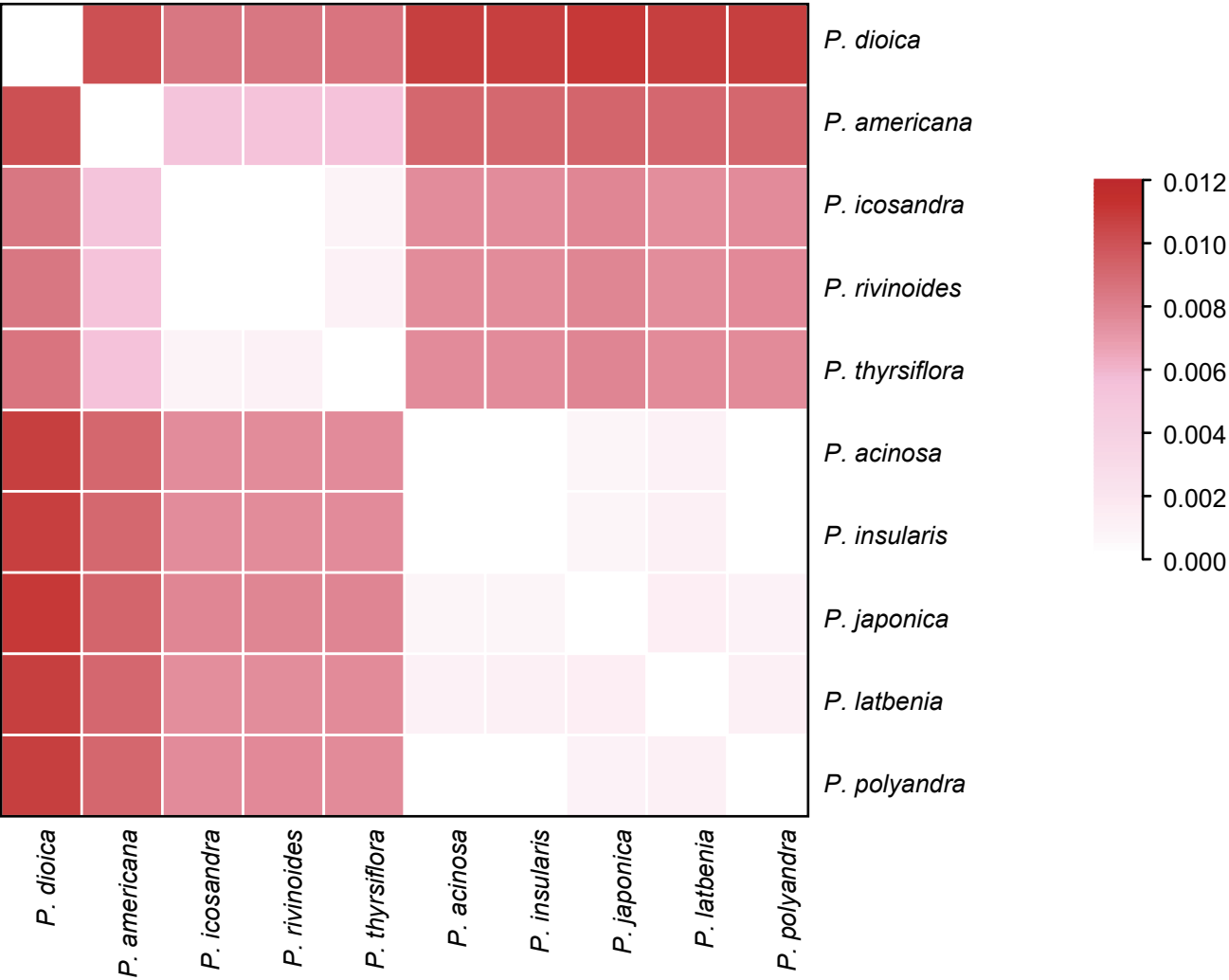

Supplement: Supplementary Figure 2 — Pairwise genetic distances among Phytolacca species. [file Data_Sheet_2.PDF]

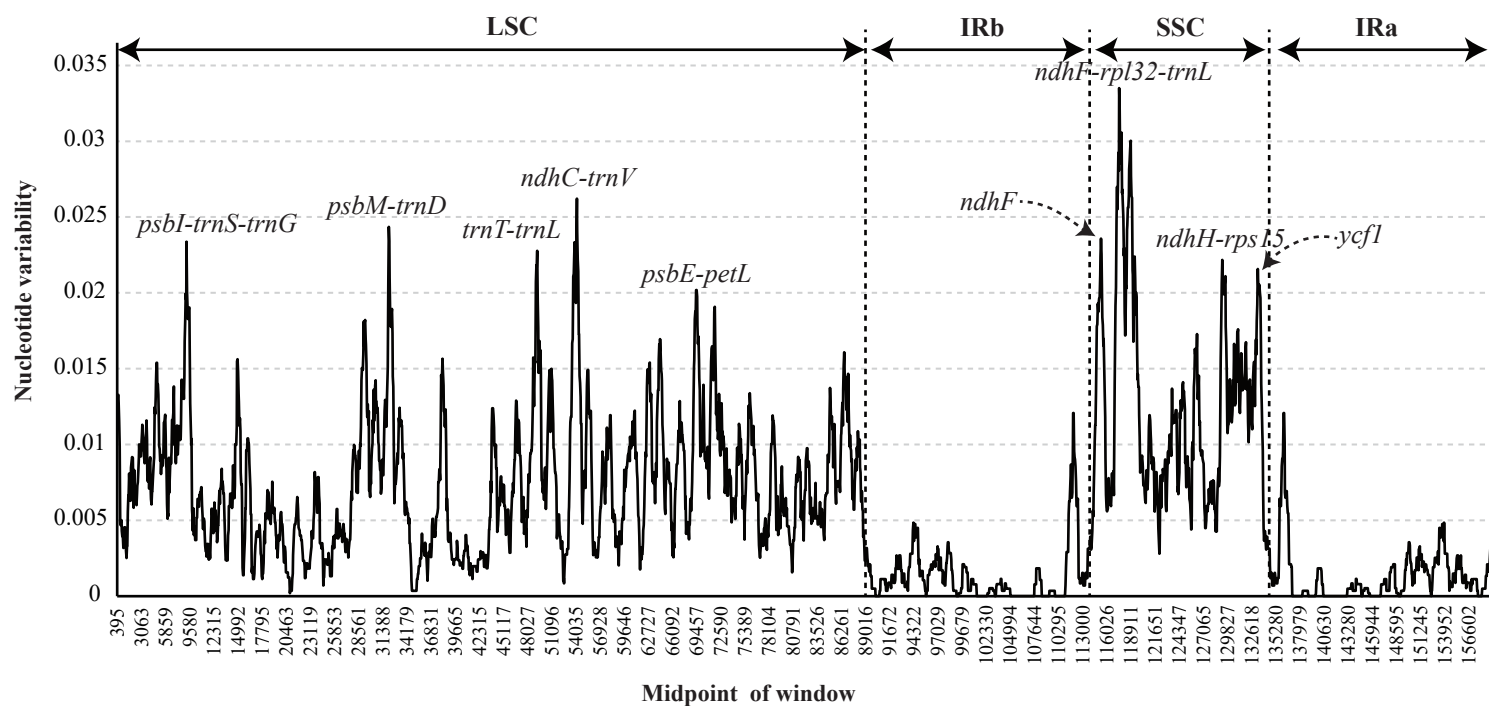

Supplement: Supplementary Figure 3 — Hypervariable regions within the chloroplast genome of Phytolacca species using sliding window analysis. Window lengths: 600 bp; step size: 50 bp. Nine regions with the highest Pi values were marked out. LSC, large single-copy region, IR, inverted repeat region, SSC, small single-copy region. x-axis: position of the midpoint of a window; y-axis: nucleotide diversity of each window. [file Data_Sheet_3.PDF]

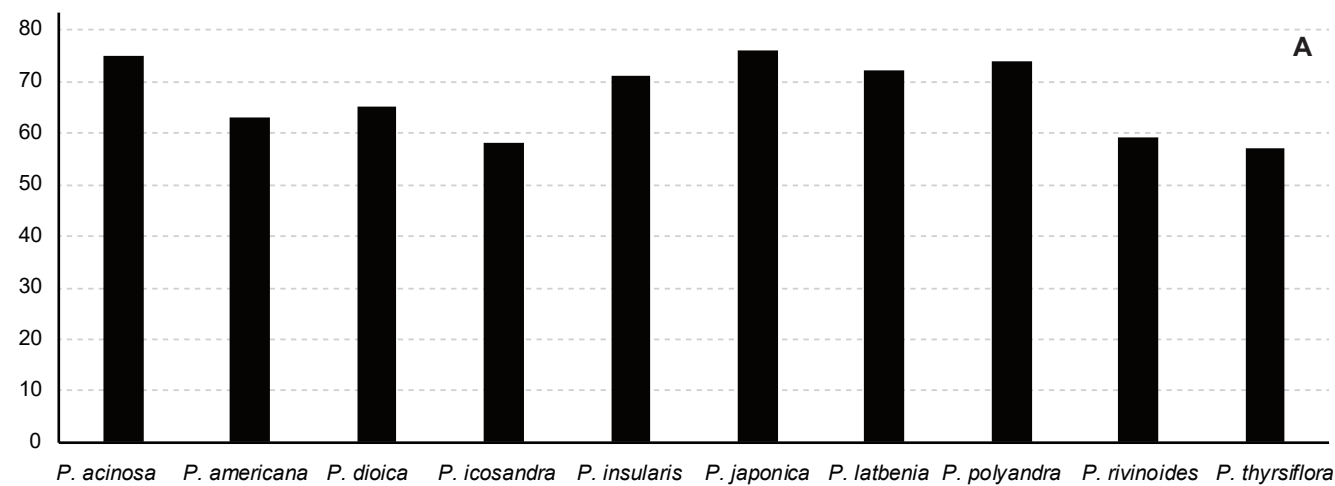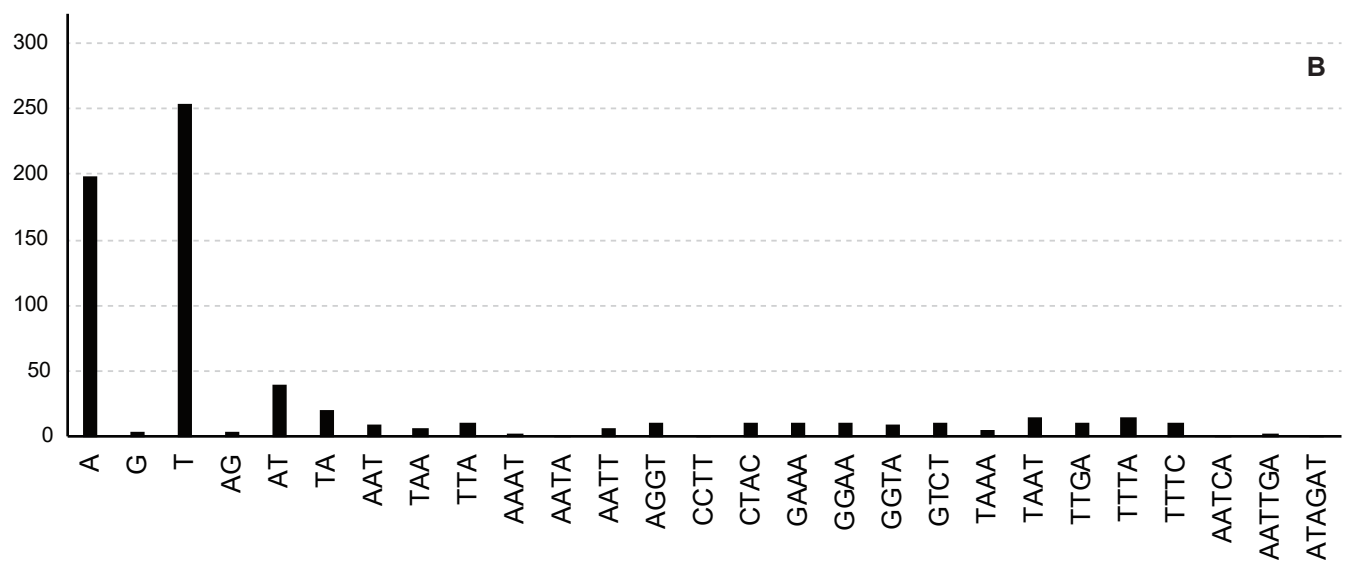

Supplement: Supplementary Figure 4 — Frequency of SSRs in the Phytolacca chloroplast genomes. (A) The number of SSRs detected in the different Phytolacca species; (B) The number of SSR types in different Phytolacca species. [file Data_Sheet_4.PDF]

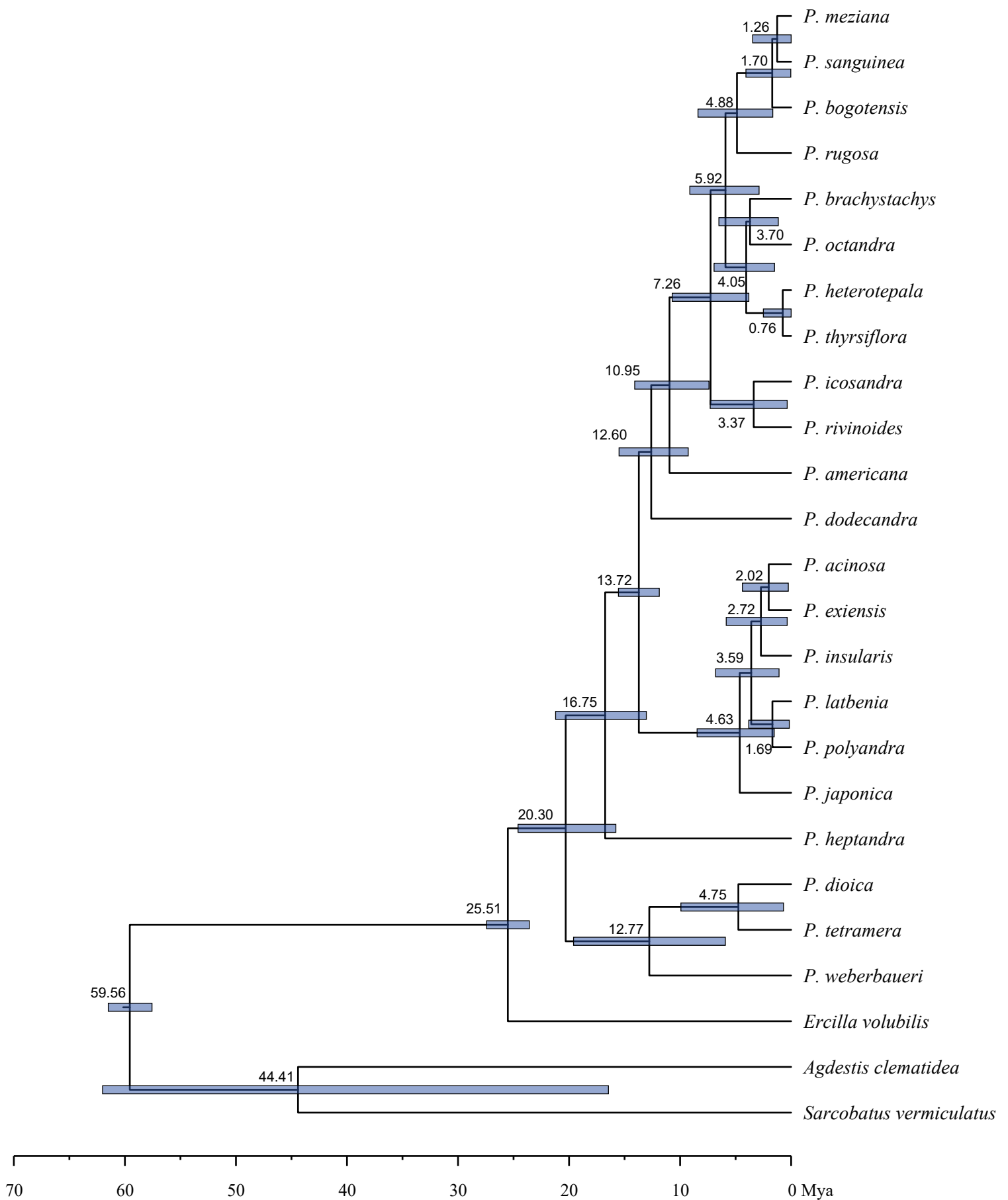

Supplement: Supplementary Figure 5 — Divergence times of Phytolacca obtained from BEAST analysis based on the 3g22s dataset. The mean divergence time of the nodes is shown next to the nodes, while the blue bars correspond to the 95% highest posterior density (HPD). [file Data_Sheet_5.PDF]

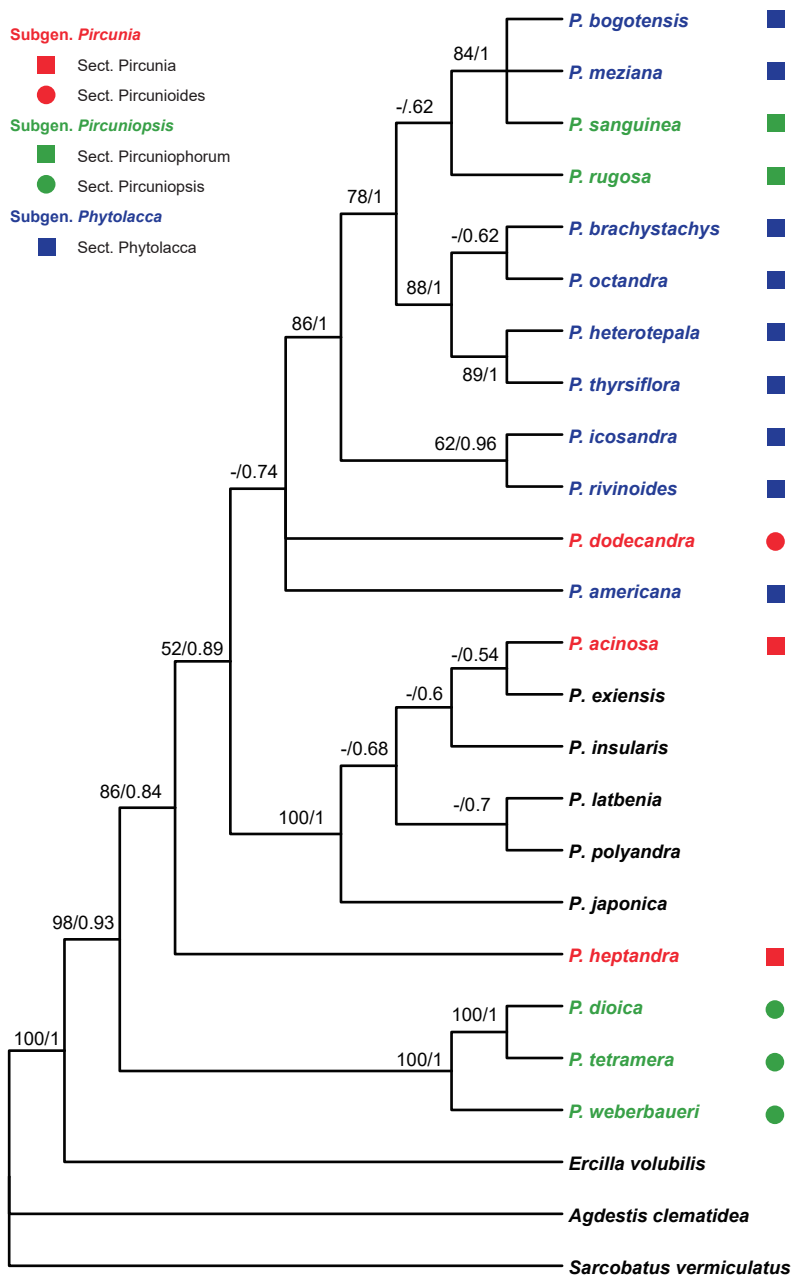

Supplement: Supplementary Figure 6 — Phylogenetic trees of Phytolacca based on the 3g22s dataset. ML bootstrap support values/Bayesian posterior probabilities were presented at each node. The infrageneric classification of the genus Phytolacca by Nowicke (1968) was mapped in the tree. The species which were not marked with the subgenus/section, were recently described or not accepted by Nowicke (1968). [file Data_Sheet_6.PDF]
