# Supplementary material for: Phylogenomics Reveals the Evolutionary History of Phytolacca (Phytolaccaceae)
Source: Front Plant Sci. 2022 Jun 10;13:844918. doi: 10.3389/fpls.2022.844918 (PMC9226614; doi:10.3389/fpls.2022.844918)
Supplement: Supplementary Figure 1 — Map of the chloroplast genome of the genus Phytolacca. [file Data_Sheet_1.PDF]

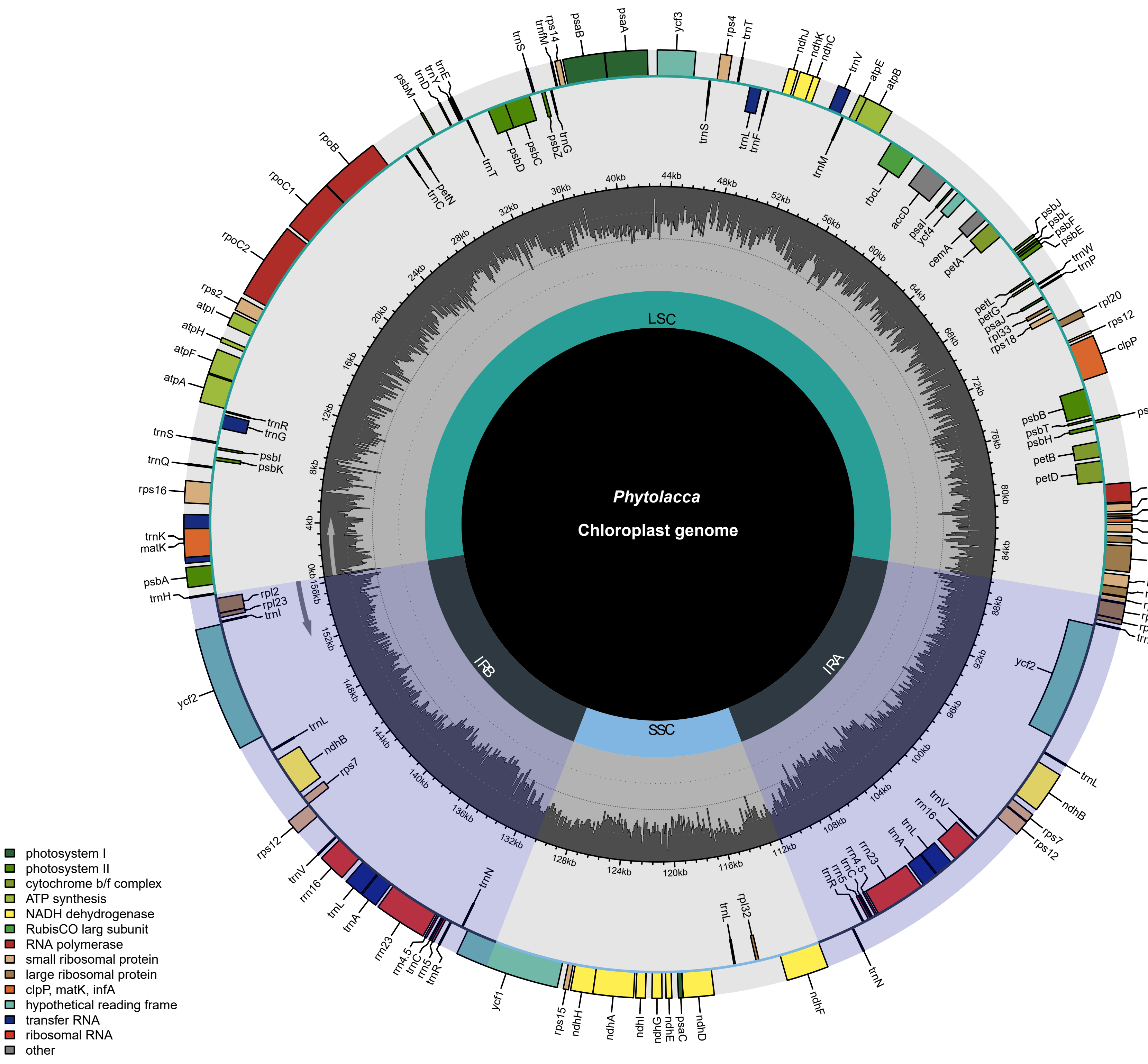

Phytolacca
Chloroplast genome
LSC
IRA
IRB
SSC
0kb
4kb
8kb
12kb
16kb
20kb
24kb
28kb
32kb
36kb
40kb
44kb
48kb
52kb
56kb
60kb
64kb
68kb
72kb
76kb
80kb
84kb
88kb
92kb
96kb
100kb
104kb
108kb
112kb
116kb
120kb
124kb
128kb
132kb
136kb
140kb
144kb
148kb
152kb
156kb
trnS
trnfM
rps14
psaB
psaA
ycf3
rps4
trnT
ndhJ
ndhK
ndhC
trnV
atpE
atpB
trnS
trnL
trnF
trnM
rbcL
accD
psaI
ycf4
cemA
petA
psbJ
psbL
psbF
psbE
trnW
trnP
petL
petG
psaJ
rpl33
rps18
rpl20
rps12
clpP
psbB
psbT
psbH
psbN
petB
petD
rpoA
rps11
rpl36
infA
rps8
rpl14
rpl16
rps3
rpl22
rps19
rpl2
rpl23
trnI
ycf2
trnL
ndhB
rps7
rps12
trnV
rrn16
trnI
trnA
rrn23
rrn4.5
rrn5
trnR
trnN
rpl32
trnL
ndhF
ndhD
psaC
ndhE
ndhG
ndhI
ndhA
ndhH
rps15
ycf1
trnN
rrn23
rrn4.5
trnC
rrn5
trnR
trnA
trnL
rrn16
trnV
rps12
rps7
ndhB
trnL
ycf2
trnI
rpl23
rpl2
trnH
psbA
matK
trnK
rps16
trnQ
psbK
psbI
trnS
trnG
trnR
atpA
atpF
atpH
atpI
rps2
rpoC2
rpoC1
rpoB
trnC
petN
psbM
trnD
trnY
trnE
trnT
psbD
psbC
psbZ
trnG
photosystem I
photosystem II
cytochrome b/f complex
ATP synthesis
NADH dehydrogenase
RubisCO larg subunit
RNA polymerase
small ribosomal protein
large ribosomal protein
clpP, matK, infA
hypothetical reading frame
transfer RNA
ribosomal RNA
other
